# Case of Hydatids in the Tibia, in Which Four Inches of the Anterior Part of the Bone Were Removed

**Published:** 1827-06

**Authors:** W. J. Wickham

**Affiliations:** Winchester Hospital.


*Case of Hydatids in the Tibia, in which four inches of the anterior part of the Bone were removed.* By W. J. Wickham, jun. Esq. (Winchester Hospital.)

If you deem the subjoined case worthy of a place in your Journal, you will oblige me by giving it insertion. It appeared in the Lancet of a few weeks back, without my sanction or authority; but the case, as it is there communicated, being incomplete, I do not hesitate to give it publicity through your Journal.

This case I believe to be of very uncommon occurrence, and I know of no recorded history of the same disease; though I understand Sir A. Cooper has in his possession a humerus, the interior of which was occupied by hydatids: the bone was fractured, and in consequence the limb amputated.

Elizabeth Stanbrook, ætatis thirty-five, of healthy constitution, was the subject of the case I have alluded to. On the 20th of September, 1825, whilst walking in the park at Stratton, and suddenly turning round, she felt her left leg give way, with a snapping noise, and, on falling down, discovered that her leg was broken. My father, who visited her soon after the accident, placed the limb in the usual position, and packed it in splints; ordering a saturnine application to be constantly used, in order to subdue the tumefaction which had come on opposite the fracture.

Upon inquiry into the history of the disease, under the conviction that some local or constitutional cause had given rise to the fracture, the following statement was reported:—About six years previous to the accident, she had been cut with a scythe, the point of which had penetrated the bone, but the wound soon healed. Soon after this, a tumor formed on the part, and continued gradually to increase till it had acquired the size of a hen's egg. It had been painful, and appeared to weaken the leg for some time previous to the accident.

As soon as the inflammatory swelling which was caused by the injury had subsided, I made an examination of the chronic tumor, which was soft and compressible, and its contents could be emptied into the body of the bone; but, on taking off the pressure, the swelling regained its usual size. The fractured ends of the bone were ragged, indicating considerable disease. The limb was packed as before in splints, and kept at rest for three months, at the end of which time no union was effected; when the poor woman consented to undergo an operation for the evacuation of the tumor, and the removal of the diseased bone, which, though proposed to her, she would not agree to before this time. She therefore came into the hospital.

On the 7th of January, 1826, the operation was performed as follows:—An incision was made, about six inches in length, on the face of the tibia, and into the tumor; upon which a number of small hydatids escaped. It was now found that the whole interior of the bone was filled with hydatids, which varied from the size of a small shot to that of a marble. These were all taken away, to the amount of a teacup full. The fracture of the bone was transverse, the edges of the fractured pieces very unequal; and a shell of bone only remained for about an inch above and below the fracture, and so thin that a very slight injury would have broken it. About four inches of the fore part of the tibia were removed, which included the rough edges. The limb was now laid in splints, and treated as a compound fracture. The wound granulated, and healed rapidly under the usual treatment.

At this time the woman has sufficient strength of the limb to sustain the weight of her body, and enable her to follow her usual occupations for some time in the day. The limb is as straight as after any case of fracture, and not shorter than the other. The constitution is improved, and the poor woman again pregnant.

The singularity of this disease has less induced me to offer it to public notice than its practical application. It well proves how much bone may be exposed and destroyed, and again generated.

In this case, about four inches of the anterior part of the tibia were taken away, the whole cavity exposed, and the bone fractured; yet a firm and useful limb has been restored.

I may here mention that a great number of cases of caries and necrosis have occurred to my colleagues and myself in the hospital, in which extensive portions of bone have been removed with the happiest effect, and limbs saved where I believe it is a very general practice to amputate.*

*Winchester; April 7th,* 1827.

### CRITICAL ANALYSES.

* Our readers will find a case of Hydatids between the Tables of the Skull, by Mr. KEATE, in the tenth volume of the Med.-Chirurg. Transactions.

